# Relaparoscopic Treatment of Recurrences after Previous Laparoscopic Inguinal Hernia Repair

**DOI:** 10.1155/2013/260131

**Published:** 2013-11-28

**Authors:** Metin Ertem, Volkan Ozben, Hakan Gok, Emel Ozveri

**Affiliations:** ^1^Department of General Surgery, Istanbul University, Cerrahpasa Medical Faculty, Cerrahpasa, Fatih, 34098 Istanbul, Turkey; ^2^General Surgery Clinic, Kozyatagi Acibadem Hospital, 34742 Istanbul, Turkey

## Abstract

*Background*. Relaparoscopic treatment of inguinal hernia recurrences has become a relatively new concept with favourable results. The purpose of this study was to examine a series of relaparoscopic repair, present technical experiences, and the clinical outcomes in this subset of patients. *Patients and Methods*. The medical records of five patients who underwent relaparoscopic repair (TAPP or TEP) for a recurrence between March 2005 and September 2012 were retrospectively reviewed. *Results*. All the patients were male with a mean age of 45 years. Technical failures in the previous repairs were the main factors contributing to recurrences. In two re-TEP cases with no previous mesh fixation, the old mesh remained on the peritoneal side during preperitoneal dissection and this greatly facilitated surgical manipulation. The mean operative time was 93 min (range, 45–120 min). There were no conversions, no intraoperative complications, and no morbidity or rerecurrence after a mean follow-up period of 17 months (range, 7–24 months). *Conclusion*. Relaparoscopic repair appears to be safe and effective in the treatment of recurrent inguinal hernia and repeated TEP could be a simpler approach than expected in the presence of no prior mesh fixation.

## 1. Introduction

Inguinal hernia repair is one of the most frequently performed operations in general surgery. With the introduction of laparoscopy in hernia surgery in 1990s, laparoscopic posterior repair (transabdominal preperitoneal (TAPP) and totally extraperitoneal (TEP)) has gained increasing popularity and emerged as the procedure of choice over open conventional techniques due to its well-established advantages such as lower rates of postoperative pain, rapid return to normal activities, and a lower incidence of infections. The major concern after inguinal hernia repair is recurrence. Recurrence rate after laparoscopic repair is comparable to that of open conventional techniques; however, such recurrences do occur after a laparoscopic repair with a reported rate of up to 5% [1, 2]. It is recommended that anterior mesh repair be performed for a recurrent hernia after previous posterior repair due to the increased risk of complications associated with the repeated posterior repair [3]. However, repeated laparoscopic treatment of hernia recurrences after previous posterior repair has become a relatively new concept and data on an increasing number of reported series has shown promising results with this approach in terms of safety, feasibility, and reliability [4–11].

We performed the first laparoscopic inguinal hernia repair in 1993 and since then we have widely employed this approach in the treatment of both primary and recurrent inguinal hernias. The purpose of this study was to examine a series of relaparoscopic surgeries for recurrences after previous laparoscopic inguinal hernia repair, present technical experiences, and the clinical outcomes in this subset of patients.

## 2. Patients and Methods 

Between March 2005 and September 2012, five patients underwent relaparoscopic repair (TAPP or TEP) for a recurrence after previous laparoscopic inguinal hernia repair at Istanbul University Cerrahpasa Medical School and Acibadem Kozyatagi Hospitals. The medical records of these patients were prospectively entered to a database and the data were retrospectively reviewed. All the patients had been initially treated in outside medical centers and then referred to us for a definitive treatment for recurrences. All the recurrences were detected by both physical examination and ultrasonography.

Written informed consent was taken from each patient after the patients were informed of the details of the relaparoscopic procedure. The medical records were analyzed to document patient demographic features, types of previous hernia, primary procedure, etiology of recurrences as detected intraoperatively, types of re-laparoscopic procedure, operative time, complications, conversions, and postoperative course (hospital stay, outpatient followup and rerecurrences).

### 2.1. Operative Technique

In our early experience with relaparoscopic repair, we used the TAPP technique for the treatment of recurrent hernias. Subsequently, with our increasing experience in the TEP technique, this approach has been preferred for the treatment of such recurrences.

**Figure 1:**
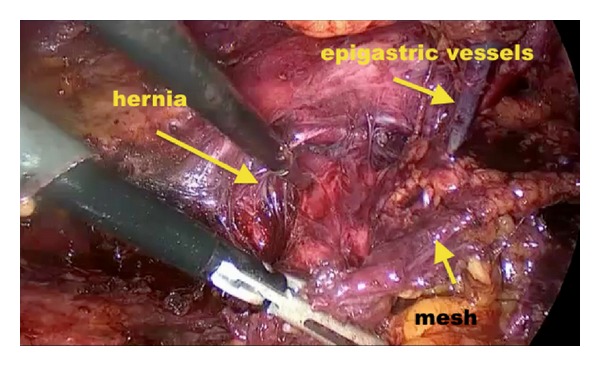
Intraoperative view of the previously placed mesh.

**Figure 2:**
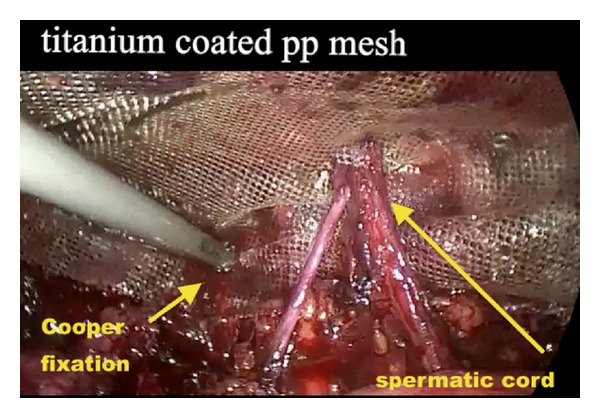
Placement of a new mesh.

The repeated TAPP and TEP repairs were performed in a standard fashion. Overall, the techniques we employed were similar to those which were previously described by van den Heuvel and Dwars [11] for TAPP and Ferzli and Khoury [10] for TEP. In short, the three-port technique was routinely employed in both techniques under general anesthesia using the three previous trocar incisions. In the TAPP repair, the peritoneum was mobilized transabdominally above the hernial defect and meticulous blunt and sharp dissection was carried out to separate the adhesions from the old mesh and the surrounding structures. In the TEP repair, blunt dissection with balloon was performed and the preperitoneal space was insufflated with carbon dioxide. The plain between the mesh and the abdominal wall was dissected and all potential hernia defects were carefully exposed (Figure 1). The anatomical landmarks (Cooper's ligament, the iliopubic tract, and inferior epigastric vessels) were identified and the etiology and type of the recurrent hernia were noted. In the presence of mesh migration or shrinkage, attempts were made to remove the old mesh. After adequate space was created around the cord structures, a 15 × 10 cm polypropylene mesh was placed (over the old mesh if not removed) to reinforce the myopectineal orifice. The mesh was prepared with a slit from its lateral edge and fixation was routinely performed on the pubic bone, Cooper's ligament, and the aponeurotic arch with tacks. The free lower lateral leg of the mesh was passed under the cord, the two legs were overlapped and then anchored to each other at the lateral edge with tacks, giving the mesh a conical shape (Figure 2). Following desufflation, the trocar sites were closed in a usual manner. No drains and no Foley catheters were placed in any patient.

On discharge, patients were instructed to wear suspensory underpants for 10 days and any strenuous physical exercise was discouraged during the first postoperative month. All the patients were visited and physically examined at the outpatient clinic after 10 days, third month, first year, and subsequently on an as-needed basis.

## 3. Results 

All the five patients were male with a mean age of 45 years (range, 32–54 years). Patient demographic data, hernia characteristics, and operative features are detailed in Table 1.

The previous techniques used were the TAPP in three patients and TEP in two patients. All the recurrences were on the same side. One patient previously had had one open and one laparoscopic hernia repairs due to rerecurrence (case 1). The mean interval between the first laparoscopic and the relaparoscopic repairs was 8 months (range, 1–13 months). Technical problems such as insufficient mesh size, mesh migration, and insufficient fixation were the main factors contributing to recurrences. During the relaparoscopic repair, placement of a new mesh (with or without removal of the old mesh) and fixation were performed in all the patients. In two cases with no previous mesh fixation (cases number 4 and 5), the old mesh remained on the peritoneal side during preperitoneal dissection in re-TEP repairs and this greatly facilitated surgical manipulation.

The mean operative time was 93 min (range, 45–120 min). There were no conversions or intraoperative complications in any of the cases. In order to remove the old mesh in one case with mesh shrinkage (case number 3), the inferior epigastric artery had to be ligated due to tight adhesions between the mesh and the artery. In this case, peritoneal tear also occurred; however, it did not obscure the operative field.

All the patients were discharged from hospital on the first postoperative day. Seroma formation occurred in two patients (cases number 1 and 2). The sizes of the seroma, as predicted on physical exam, were approximately 5 × 3 cm and 4 × 3 cm in cases 1 and 2, respectively. As a conservative management, both seromas were allowed to resolve by itself without necessitating needle aspiration or any other interventional procedures. The mean follow-up period was 17 months (range, 7–24 months). During followup, no documented case of chronic groin pain, sexual dysfunction, mesh infection, or rerecurrence were encountered.

## 4. Discussion

Today, conventional open inguinal hernia repair is the preferred method by most surgeons for the treatment of recurrences after previous laparoscopic repair and this concept is also supported by European Hernia Society [3]. However, a number of published studies have appeared in the literature addressing the use of relaparoscopic repair (TAPP or TEP) of recurrences after previous laparoscopic repair and their findings indicate that there is a place for relaparoscopic surgery in the treatment of such recurrences [4–11]. van den Heuvel and Dwars [11] and Knook et al. [5] reported on 49 and 18 TAPP repairs for recurrences after previous TAPP or TEP, respectively, and concluded that the TAPP repair is safe and reliable for recurrences. Also, Ferzli et al. [9] reported on 20 cases and found that TEP repair of recurrent inguinal hernia after a primary TEP repair is entirely feasible technically as well as entirely safe.

The repeated laparoscopic approach is considered to be more difficult and is associated with an increased risk of complications due to the distorted anatomy. Since its introduction in the 1990s, laparoscopic inguinal hernia repair has become the procedure of choice in our surgical practice and over a period of 20 years; we have gained a considerable experience and a thorough understanding of the posterior inguinal anatomy in both TAPP and TEP techniques. Eventually, in addition to repairing primary hernias, we have also employed these procedures in the aforementioned five patients for the treatment of recurrences after previous laparoscopic repair. Of note, our laparoscopic approach to such recurrences does not vary greatly from our approach for the treatment of primary inguinal hernias.

Our observations in this small series confirm the evidence that recurrences after previous laparoscopic inguinal hernia repair are mainly due to technical errors and eventually they occur early [11–14]. The recurrences were noted within a mean period of 8 months after the primary repair and these were either due to small mesh size, mesh migration; or insufficient fixation. Therefore, we believe that in addition to a proper-sized new mesh placement, mesh fixation should be performed in all such cases in order to prevent rerecurrences. The mesh should be properly placed to the inguinal floor. To achieve this, we first anchor the mesh to just over the pubic bone and Cooper's ligament with tacks and then overlap its free lateral legs around the cord with further tacks, giving the mesh a conical shape. This mesh configuration perfectly fits the anatomy of the inguinal floor, which may decrease the rerecurrence risk.

TAPP appears to be the preferred approach by some surgeons for a recurrent inguinal hernia after the previous laparoscopic repair [4, 11]. Indeed, repeated TEP repair seems to be a daunting task due to the presence of adhesions in the preperitoneal space and the scarring between the previously placed mesh and the abdominal wall. Surprisingly, in our experience with two re-TEP repairs, we observed that the previously placed mesh was mobilized easily from the abdominal wall and it remained on the peritoneal side during preperitoneal dissection and this consequently facilitated the surgical manipulation in this area. In the primary repair in these two cases, no mesh fixation had been performed and this could explain the easy mobilization of the old mesh with the peritoneum. We therefore assume that in the presence of no prior mesh fixation, which can also be demonstrated on preoperative radiologic imaging, a repeated TEP repair could be a simpler approach than expected. On the other hand, if mesh fixation was previously performed, we recommend the use of a TAPP approach due to the risk of peritoneal tear which may complicate the TEP repair. However, further experience is needed to confirm these assumptions.

There were no intra- or postoperative complications in this series. In the case where inferior epigastric artery had to be ligated due to dense adhesions (case number 3), no adverse postoperative outcome was noted. Of note, case 1 did not receive mesh removal whereas case 3 did. The difference between these two cases was that in case 3, the previously placed mesh was found to be shrunken and tightly adherent to the inferior epigastric artery. In addition to this, mesh migration was also seen and the mesh could be palpated from the skin externally. That is why the mesh had to be removed in this case. In case 1, however, the old mesh was just small and it was not removed since it did not complicate the relaparoscopic surgery.

One major concern is the rerecurrence since the risk of recurring increases every time a hernia recurs and surgery is repeated. No recurrence after a mean followup of 17 months in this series is in accordance with the favourable results of earlier studies [5, 9, 11]. In their larger series with a longer follow-up period, van den Heuvel and Dwars [11] and Knook et al. [5] performed 49 and 18 TAPP repairs for recurrences after previous TAPP or TEP repairs, respectively, and encountered no rerecurrences. Similarly, Ferzli et al. [9] reported on 12 TEP repairs performed for the same-sided recurrence after primary TEP and there was also no rerecurrence in this series.

Based on our experience with a small number of patients so far, relaparoscopic repair (either TAPP or TEP) appears to be a safe and effective procedure for the treatment of recurrent inguinal hernia, and repeated TEP could be a simpler approach than expected in the presence of no prior mesh fixation.

## Figures and Tables

**Table 1:** Patient demographic data, hernia characteristics, and operative features.

Patient number	Age	Position and type of primary hernia	Primary repair	Etiology of recurrent hernia	Type of recurrent hernia	Final repair
1	54	Right, direct	TAPP	Small mesh	Direct	TAPP (without old mesh removal)
2	50	Left, indirect	TAPP	Mesh migration, fixation failure	Indirect	TAPP (with partial old mesh removal)
3	52	Right, indirect	TAPP	Small mesh	Indirect	TEP (with old mesh removal)
4	32	Right, direct	TEP	Missed hernia, no fixation	Indirect	TEP (without old mesh removal)
5	35	Right, direct	TEP	Small mesh, no fixation	Direct	TEP (without old mesh removal)

## References

[1] Liem MS, van Duyn EB, van der Graaf Y, van Vroonhoven TJ (2003). Recurrences after conventional anterior and laparoscopic inguinal hernia repair: a randomized comparison. *Annals of Surgery*.

[2] Langeveld HR, Van't Riet M, Weidema WF (2010). Total extraperitoneal inguinal hernia repair compared with lichtenstein (the level-trial): a randomized controlled trial. *Annals of Surgery*.

[3] Simons MP, Aufenacker T, Bay-Nielsen M (2009). European Hernia Society guidelines on the treatment of inguinal hernia in adult patients. *Hernia*.

[4] Felix E, Scott S, Crafton B (1998). Causes of recurrence after laparoscopic hernioplasty: a multicenter study. *Surgical Endoscopy*.

[5] Knook MT, Weidema WF, Stassen LPS, van Steensel CJ (1999). Laparoscopic repair of recurrent inguinal hernias after endoscopic herniorrhaphy. *Surgical Endoscopy*.

[6] Tamme C, Scheidbach H, Hampe C, Schneider C, Köckerling F (2003). Totally extraperitoneal endoscopic inguinal hernia repair (TEP): results of 5,203 hernia repairs. *Surgical Endoscopy and Other Interventional Techniques*.

[7] Chowbey PK, Bandyopadhyay SK, Sharma A, Khullar R, Soni V, Baijal M (2003). Recurrent hernia following endoscopic total extraperitoneal repair. *Journal of Laparoendoscopic and Advanced Surgical Techniques A*.

[8] Richards SK, Vipond MN, Earnshaw JJ (2004). Review of the management of recurrent inguinal hernia. *Hernia*.

[9] Ferzli GS, Shapiro K, DeTurris SV (2004). Totally extraperitoneal (TEP) hernia repair after an original TEP: is it safe, and is it even possible?. *Surgical Endoscopy and Other Interventional Techniques*.

[10] Ferzli GS, Khoury GE (2006). Treating recurrence after a totally extraperitoneal approach. *Hernia*.

[11] van den Heuvel B, Dwars BJ (2013). Repeated laparoscopic treatment of recurrent inguinal hernias after previous posterior repair. *Surgical Endoscopy*.

[12] Leibl BJ, Schmedt C, Kraft K, Ulrich M, Bittner R (2000). Recurrence after endoscopic transperitoneal hernia repair (TAPP): causes, reparative techniques, and results of the reoperation. *Journal of the American College of Surgeons*.

[13] Knook MT, Weidema WF, Stassen LPS, van Steensel CJ (1999). Endoscopic total extraperitoneal repair of primary and recurrent inguinal hernias. *Surgical Endoscopy*.

[14] Phillips EH, Rosenthal R, Fallas M (1995). Reasons for early recurrence following laparoscopic hernioplasty. *Surgical Endoscopy*.

